# New filamin A gene mutation identified in X‐linked pediatric intestinal pseudo‐obstruction: A case report

**DOI:** 10.1002/jpr3.70277

**Published:** 2026-11-03

**Authors:** Leonor Belfond, Catheline Vilain, Helena Reusens, Anna Amoruso, Lea Boulvain, Kallirroi Kotilea, Ebru Surgun

**Affiliations:** ^1^ Department of Integrated Pediatrics and Adolescent Medicine Hôpital Universitaire des Enfants Reine Fabiola (HUDERF) Brussels Belgium; ^2^ Department of Pediatric Genetics Hôpital Universitaire des Enfants Reine Fabiola (HUDERF) Brussels Belgium; ^3^ Department of Pediatric Surgery Hôpital Universitaire des Enfants Reine Fabiola (HUDERF) Brussels Belgium; ^4^ Neonatal Intensive Care Unit, Hôpital Universitaire des Enfants Reine Fabiola (HUDERF) Brussels Belgium; ^5^ Department of Pediatric Gastroenterology, Hepatology and Nutrition Hôpital Universitaire des Enfants Reine Fabiola (HUDERF), Hopital Université de Bruxelles (HUB) Brussels Belgium; ^6^ Department of Integrated Pediatrics and Adolescent Medicine Hôpital Universitaire des Enfants Reine Fabiola Brussels Belgium

**Keywords:** chronic intestinal pseudo‐obstruction, FLNA, X‐linked PIPO

## Abstract

Pediatric intestinal pseudo‐obstruction (PIPO) represents a severe gut motility disorder leading to digestive complications with high morbidity and mortality. Neonates and young children present symptoms mimicking intestinal occlusion, leading to surgery and long‐term feeding support. The multifactorial etiologies of PIPO make the diagnosis particularly challenging. Most cases are congenital, with only a few genetic forms. Recent genetic research has identified mutations, including those in the filamin A gene (FLNA), contributing to primary X‐linked PIPO and associated syndromes. FLNA deficiency includes abnormal neuronal migration, cardiac disorders, and digestive problems ranging from severe constipation to chronic intestinal pseudo‐obstruction. We report the case of an 8‐day‐old newborn diagnosed with X‐linked PIPO due to a new FLNA mutation who did not present other associated syndromic phenotypes.

## 1 INTRODUCTION

Pediatric intestinal pseudo‐obstruction (PIPO) is a rare gastrointestinal disorder characterized by symptoms of intestinal obstruction without any mechanical blockage. It results from abnormalities of the enteric neuromuscular system, causing inefficient or absent peristalsis.^1^ The incidence is 29 per 100,000 newborns and young children.^2^ This pathology is associated with high morbidity and mortality due to severe digestive complications and nutritional deficiencies, frequently requiring parenteral nutrition (PN).

According to the international consensus published by the European Society of Pediatric Gastroenterology, Hepatology and Nutrition (ESPGHAN),^1^ PIPO diagnosis requires evidence of small intestinal neuromuscular involvement, including manometry, histopathology, or transit studies; persistent small intestinal dilation with air‐fluid levels; genetic or metabolic abnormalities and inability to maintain adequate nutrition through oral feeding. At least two of these four criteria must be met, with symptoms persisting for at least 2 months from birth or a minimum of 6 months thereafter.

Up to 80% of cases are congenital, with neuropathic forms accounting approximately two‐thirds and myopathic forms one‐third.^1^ Familial forms of PIPO may be inherited in an autosomal dominant, autosomal recessive, or X‐linked manner. Mutations responsible for PIPO have been identified only in the 21st century,^3^ enabling the diagnosis of pediatric syndromes associated with intestinal pseudo‐obstruction.

This article reports the case of a newborn diagnosed with a novel filamin A gene (FLNA) mutation responsible for X‐linked PIPO, contributing to the understanding of the disease and improving patient management.

## 2 CASE REPORT

### 2.1 Presentation

An 8‐day‐old male neonate was referred to the neonatal intensive care unit because of recurrent bilious vomiting, severe abdominal distension, and lethargy. Born at 36 weeks and 4 days, he weighed 2830 g at birth. Neonatal adaptation was uncomplicated and neonatal screening tests were normal. Postnatal echocardiography revealed an ostium secundum atrial septal defect (4–5 mm) with a good prognosis.

The patient was the first child of a non‐consanguineous couple. Maternal family history included gastroesophageal reflux disease, chronic diarrhea, irritable bowel syndrome, and lactose intolerance in the father's family.

On admission, the newborn had lost 15% of his birth weight. Abdominal radiography revealed a double‐bubble sign with intestinal dilatation and ultrasonography suggested intestinal malrotation (IM) (Figure 1). No signs of volvulus or enterocolitis were present.

**Figure 1:**
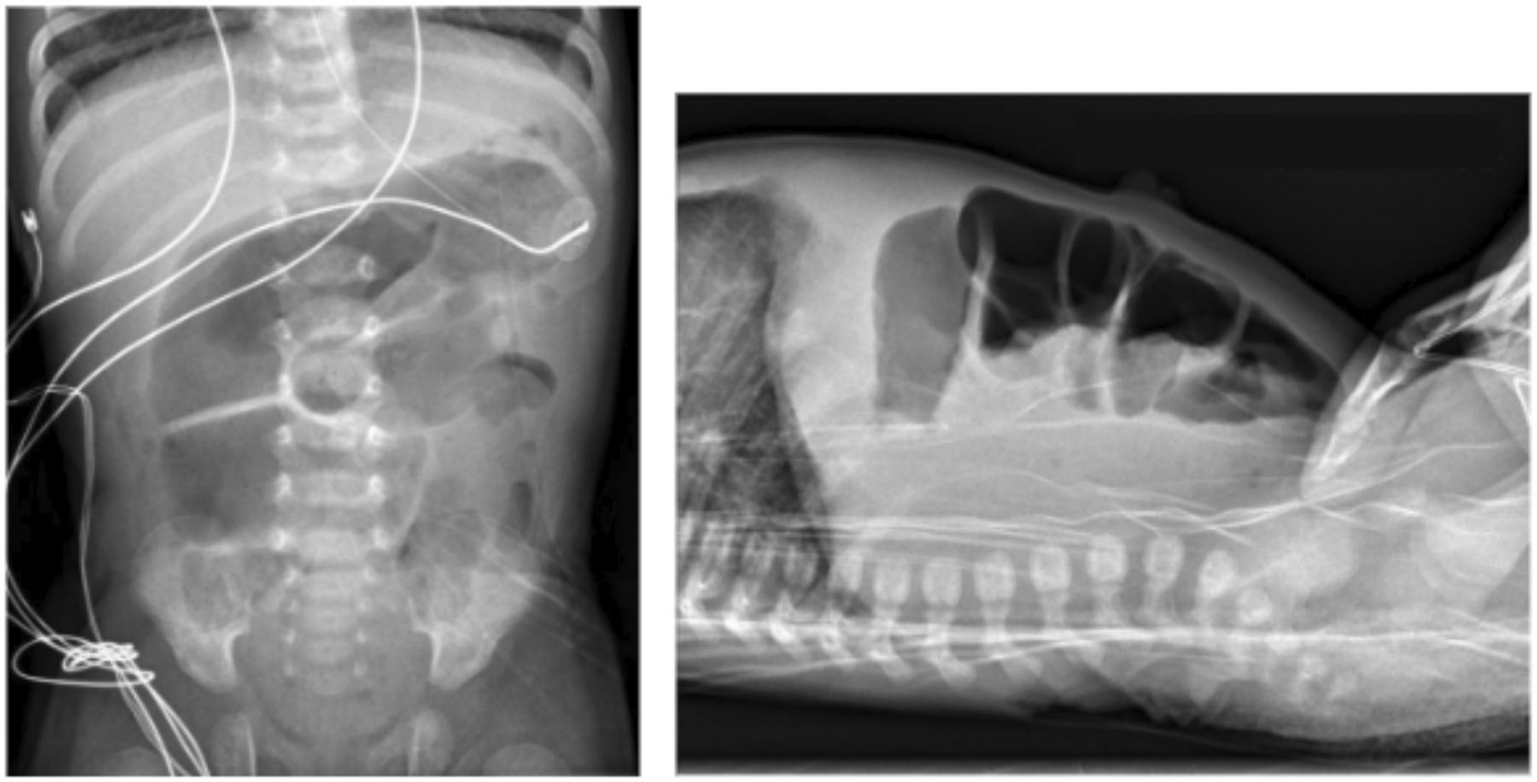
Eight‐days‐old male abdominal X‐ray showing a double‐bubble sign with intestinal dilatation.

Despite exclusive PN and gastric aspiration, there was no clinical improvement. A Ladd procedure and appendectomy were performed at 11 days of age, revealing a short bowel (70 cm). Suction rectal biopsy and appendix analysis confirmed ganglion cells, ruling out Hirschsprung disease and colonic aganglionosis.

Postoperatively, enteral feeding intolerance persisted with ongoing bilious vomiting and intestinal distension. A lower gastrointestinal opacification showed a dilated proximal bowel and a small‐caliber descending colon. At 7 weeks, a terminal ileostomy was performed with staged biopsies from small bowel and colon. Histopathologic examination (Figure 2) confirmed the presence of ganglion cells in submucosal and myenteric plexuses, excluding neural dysplasia.

**Figure 2:**
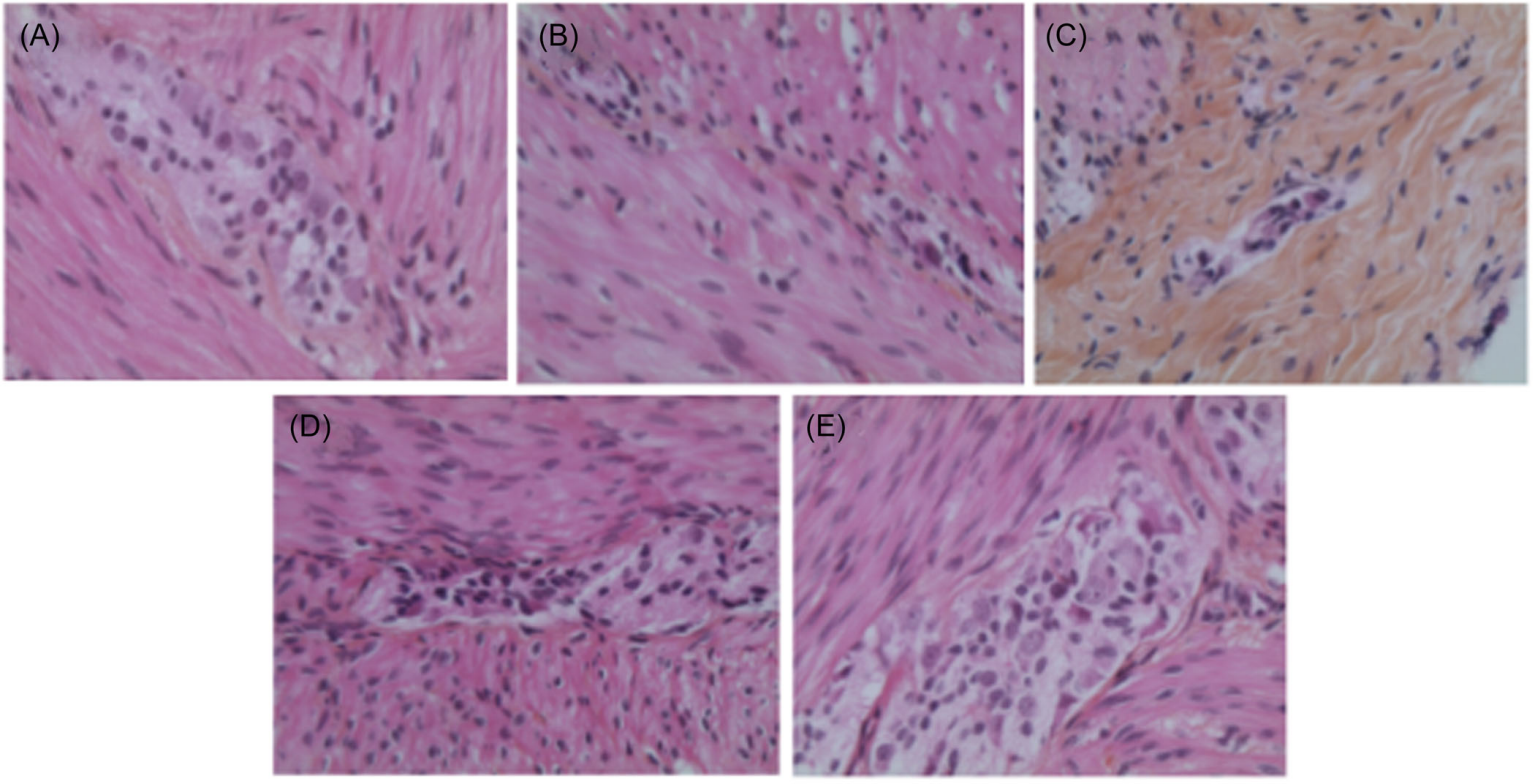
Histopathology of staged gastrointestinal biopsies. Higher‐power H&E section demonstrates Meissner's and Auerbach's plexuses containing normal‐looking ganglion cells (H&E, ×200). H&E, hematoxylin and eosin.

At 4.5 months, due to an incarcerated prolapse of the distal stoma, an early restoration of digestive continuity was performed. Additional rectal biopsies were taken and returned normal. Persistent small bowel dilation led to a Santulli Y‐shaped ileostomy at 7 months. Nutritional needs were fully supported by PN. One month after the second ileostomy, gradual oral refeeding was introduced through a gastrostomy tube with good tolerance.

### 2.2 Genetic analysis

Trio analysis of a panel of 4867 disease‐associated genes was performed. Library preparation, sequencing, bioinformatic analysis, and variant interpretation have been described previously (PMID: 40592404; PMID: 34906519). Genetic analysis identified a novel hemizygous FLNA variant (NM_001110556.1:c.60delC), predicted to result in a frameshift and premature stop codon (p.Val21Serfs*37). The variant is absent from gnomAD v4.1.1 and has not been described in the literature.

It is located in the intestinal smooth muscle‐specific FLNA transcript that utilizes an alternative translational initiation site located 82 base pairs upstream of the canonical isoform (NM_001456.3), in which other loss‐of‐function variants have been associated with isolated PIPO (PMID: 32108395, PMID: 29024177). Therefore, the variant was classified as likely pathogenic based on American College of Medical Genetics and Genomics (ACMG) criteria (PVS1, PM2).

The same variant was present in the mother in a heterozygous state, corresponding to her history of digestive symptoms.

### 2.3 Follow‐up

After 9 months of hospitalization, the patient was transferred to a specialized tertiary center and returned home afterward with continued PN and partial oral feeding. He presented complications such as moderate electrolyte imbalances and PN‐associated liver dysfunction. During the first 2 years of life, he experienced multiple hospitalizations and two episodes of bacteremia related to the central venous catheter. He also experienced multiple stoma prolapses but did not require additional surgery. Currently, the patient has spontaneous bowel movements, reduced stoma leakage, and improved tolerance to oral ingestion, although he remains PN‐dependent.

## 3 DISCUSSION

X‐linked PIPO is a rare disorder caused by FLNA mutations. Two FLNA isoforms have been described in vivo studies: a long isoform expressed in intestinal smooth muscle that plays a role in motility and a short isoform predominantly expressed in other tissues including the brain.^4^, ^5^ Since the patient presents only gastrointestinal symptoms, his phenotype can be explained by a loss of function of the intestinal smooth muscle‐specific FLNA isoform. Although no extra‐intestinal manifestations are currently present, long‐term follow‐up remains needed to detect potential neurodevelopmental delays or epilepsy.

A literature review identified 40 cases of X‐linked PIPO associated with FLNA mutations, showing a broad phenotypic spectrum that includes periventricular nodular heterotopia, cardiovascular anomalies, short bowel syndrome, IM, and PIPO.^6^, ^7^

The identified variant affects the intestinal smooth muscle‐specific FLNA transcript, in which several loss‐of‐function variants have previously been associated with isolated PIPO. Although no functional studies are available for this variant, its location, predicted loss‐of‐function effect, and the concordant phenotype strongly support its pathogenicity.

## 4 CONCLUSION

This case highlights PIPO's diagnostic and management challenges, particularly in the context of novel genetic mutations. Given the severe prognosis, genetic testing should be considered in neonates with unexplained intestinal obstruction. A recent survey of 11 European centers^8^ found that while more than 90% used genetic testing routinely, only one‐third had a standard genetic panel, underscoring the need for a uniform approach in PIPO genetic testing.

## CONFLICT OF INTEREST STATEMENT

The authors declare no conflicts of interest.

## ETHICS STATEMENT

Informed consent was obtained from the parents for publication of this case report.
